# *In Silico* Analysis of the Minor Histocompatibility Antigen Landscape Based on the 1000 Genomes Project

**DOI:** 10.3389/fimmu.2018.01819

**Published:** 2018-08-16

**Authors:** Nadia A. Bykova, Dmitry B. Malko, Grigory A. Efimov

**Affiliations:** Laboratory of Transplantation Immunology, National Research Center for Hematology, Moscow, Russia

**Keywords:** allogeneic hematopoietic stem cell transplantation, minor histocompatibility antigens, alloantigens, SNP, immunopeptidome

## Abstract

Allogeneic hematopoietic stem cell transplantation (allo-HSCT) is routinely used to treat hematopoietic malignancies. The eradication of residual tumor cells during engraftment is mediated by donor cytotoxic T lymphocytes reactive to alloantigens. In a HLA-matched transplantation context, alloantigens are encoded by various polymorphic genes situated outside the HLA locus, also called minor histocompatibility antigens (MiHAs). Recently, MiHAs have been recognized as promising targets for post-transplantation T-cell immunotherapy as they have several appealing advantages over tumor-associated antigens (TAAs) and neoantigens, i.e., they are more abundant than TAAs, which potentially facilitates multiple targeting; and unlike neoantigens, they are encoded by germline polymorphisms, some of which are common and thus, suitable for off-the-shelf therapy. The genetic sources of MiHAs are nonsynonymous polymorphisms that cause differences between the recipient and donor proteomes and subsequently, the immunopeptidomes. Systematic description of the alloantigen landscape in HLA-matched transplantation is still lacking as previous studies focused only on a few immunogenic and common MiHAs. Here, we perform a thorough *in silico* analysis of the public genomic data to classify genetic polymorphisms that lead to MiHA formation and estimate the number of potentially available MiHA mismatches. Our findings suggest that a donor/recipient pair is expected to have at least several dozen mismatched strong MHC-binding SNP-associated peptides per HLA allele (116 ± 26 and 65 ± 15 for non-related pairs and siblings respectively in European populations as predicted by two independent algorithms). Over 70% of them are encoded by relatively frequent polymorphisms (minor allele frequency > 0.1) and thus, may be targetable by off-the-shelf therapeutics. We showed that the most appealing targets (probability of mismatch over 20%) reside in the asymmetric allele frequency region, which spans from 0.15 to 0.47 and corresponds to an order of several hundred (213 ± 47) possible targets per HLA allele that can be considered for immunogenicity validation. Overall, these findings demonstrate the significant potential of MiHAs as targets for T-cell immunotherapy and emphasize the need for the systematic discovery of novel MiHAs.

## Introduction

T-cell immunotherapy is extremely promising in cancer treatment, as was demonstrated in a number of successful cases of targeting tumor-associated antigens (TAAs) and/or neoantigens in melanoma and other solid cancers (1). The distinct feature of hematopoietic malignancies is that a patient’s hematopoietic system containing the malignant clone can be completely eradicated and replaced by the hematopoietic system of a donor. Allogeneic hematopoietic stem cell transplantation (Allo-HSCT) not only provides healthy blood cells, T-cell-replete graft and/or subsequent donor lymphocyte infusions also facilitate the elimination of residual disease by targeting alloantigens in the “graft versus leukemia” (GvL) reaction (2, 3). Thus, allo-HSCT can be considered a form of immunotherapy, where the targets are recipient-specific peptides. When presented in the context of matched HLA they are reffered to as minor histocompatibility antigens (MiHAs). It was suggested that MiHAs could be used as targets for leukemia relapse therapy after allo-HSCT or for relapse prophylaxis in high-risk patients alongside TAAs and neoantigens (4, 5, 6). MiHA-specific T-cell clones can be generated either by the antigen-specific expansion (7, 8), or by transducing cells with the MiHA-specific T-cell receptors (TCRs) (9, 10). However, to avoid “graft-versus-host” disease (GvHD), a severe complication after allo-HSCT, therapeutic immune response has to discriminate hematopoietic from non-hematopoietic (not affected by the disease) tissues. Ideal therapeutic MiHAs are encoded by genes exclusively or predominantly expressed in the hematopoietic lineage. Unfortunately, out of the 70 MiHAs discovered so far (11) very few are specific to the hematopoietic tissue (for review see Bleakley and Riddell (5)). It is unclear how many MiHAs remain to be discovered.

On the other hand, it was recently shown that the magnitude of response and the number of involved alloreactive clones rather than the expression pattern of the target antigen played a more prominent role in determining if GvL would be complicated with GvHD (12). Thus theoretically even ubiquitous MiHA could be safely targeted.

The genetic sources of MiHAs are nonsynonymous polymorphisms (nsSNP) that cause differences between the recipient and the donor proteomes and, subsequently, immunopeptidomes (11). MiHA-specific T cells in MiHA-negative donors are not eliminated during negative selection in the thymus, and upon transfer to MiHA-positive recipient may encounter their targets presented on recipient antigen presenting cells, subsequently become activated and drive alloreactive immune response. A similar situation but in the reverse direction arises in the case of organ transplantation (13). Most of the known MiHAs were discovered by the “forward immunology” approach, comprising the isolation of the alloreactive clone from the patient and subsequent inference of immunogenic nsSNP. Originally, cDNA libraries (14, 15) and genetic linkage analysis (16, 17) were used, while most recently genome-wide association studies were shown to be efficient (18). The major limitation of this approach is that it only allows the detection of the *in vivo* immunodominant targets. However, less immunogenic MiHAs may still be applicable for therapy, if antigen-specific clones can be generated *in vitro*. Also, the described above framework does not allow for systematic addressing of the actual MiHA landscape.

The complimentary “reverse immunology” approach is usually based on the mass spectrometry (MS) analysis of MHC-eluted peptides. The peptides detected by MS are further mapped to polymorphic genomic regions. This approach was validated by testing the immunogenicity of predicted MiHAs (19), while MHC-tetramer verification was less effective (20). Over a hundred new MiHAs, restricted by the HLA-*02:01 and HLA-B*44:03 alleles, were predicted based on MS data (19). However, there is evidence that MS may significantly underpredict the number of candidate MiHAs, due to peptide loss, both during the purification step and the peptide mapping procedure. T-cell immunogenicity assays appear to be more sensitive than MS analysis (21, 22).

On the other hand, the “reverse immunology” approach, based solely on the *in silico* prediction of MHC affinity, is thought to substantially overpredict the number of MiHA candidates. This is the result of the complexity of the antigen presentation process, which, apart from peptide binding to the MHC (this step can be relatively well predicted) includes proteasomal degradation of the proteins, TAP transport to the endoplasmic reticulum, peptide cleavage by the peptidases, and other factors. Additionally, some MHC-associated peptides and, potentially, MiHAs were shown to arise from non-coding regions (23) or as a result of a proteasomal splicing (24), which is even more challenging for the *in silico* prediction. As a result, no comprehensive description of the MiHA landscape was made. However, the recent application of *in silico* prediction to the neoantigen discovery showed remarkable performance with the substantial amount of predicted mutations confirmed as immunogenic (25, 26). In contrast to the neoantigens, all frequent nsSNPs are listed in the genomic variation databases (27), and thus the immunogenicity assessment of the most frequent polymorphisms is fundamentally feasible.

Here, we aim to advance towards the goal of the comprehensive description of the alloantigen landscape in the HLA-matched transplantation. Earlier approaches to systematically describe MiHA mismatches were based on the exome-sequencing data of the patients and the donors undergoing transplantation (28, 29). Although these studies had the advantage of using HLA and genomic data of actual transplantation pairs, the major limitation of these approaches was that they lacked the allele frequency analysis due to the small number of samples. Below we report *in silico* analysis of the public genomic data and attempt to classify the features of the immunopeptidome mismatches in virtual (*in silico* paired) donor/recipient pairs. Using the MHC binding prediction algorithms, available MS databases and the data about known MiHAs, we speculate about the total number of MiHAs in the population. The results emphasize the need for systematic immunogenicity verification of *in silico* predicted potential MiHAs.

## Materials and Methods

### Genomic Data

The reference genomic data from the ENSEMBL release 85 and Phase 3 1000 Genomes Project genome variation data were downloaded from ENSEMBL FTP site: ftp://ftp.ensembl.org. Only transcripts from autosomes that have RefSeq Protein accessions were considered. From the initial 34 127 transcripts, 50 were excluded due to the presence of stop codons inside annotated coding regions (these stop codons can be translated as stop, or selenocysteine under some conditions). Variations that extended beyond exon boundaries were ignored; the fraction of such variations was negligible. The IDs of considered transcripts and discarded SNPs for each studied pair are listed in Table S1 in Supplementary Material.

Genomic samples of related individuals from 1000 genomes project were obtained at ftp://ftp.1000genomes.ebi.ac.uk/vol1/ftp/release/20130502/supporting//related_samples_vcf, structural variants data were excluded. As the data were only available in GRCh37 coordinates, the files were converted to GRCh38 coordinates with CrossMap software (https://sourceforge.net/projects/crossmap/) using the recommended liftover file (GRCh37_to_GRCh38.chain). Less than 0.35% of total entries failed to remap. The output files were compared to the VCF files of non-related samples used in the general analysis; only the transcripts that did not differ by the number or identity (i.e., SNP genomic position and the set of allelic variants) of the variations in coding regions were used for the analysis. This procedure filtered out 5% of transcripts; the remaining transcripts used for the comparative analysis of related and unrelated individuals are listed in Table S1 in Supplementary Material.

### HLA Genotyping Data

HLA typing is available for 932 samples from the 1000 Genomes Project (30). The data were downloaded from http://www.internationalgenome.org/category/hla/. These data were used to compare genetic differences in HLA-matched and randomly selected HLA-unmatched pairs. For European population 4 HLA-matched pairs were available; their HLA genotype is listed in Table S1 in Supplementary Material.

### MiHA Prediction

The minor histocompatibility antigen prediction for the allo-HSCT donor-recipient pair consists of two steps: (1) identification of peptides that can arise in the process of proteasomal degradation in the recipient, but not in the donor (these peptides are addressed in this paper as “unique recipient peptides,” URPs) and (2) estimation of the probability of their presentation on the surface of the recipient’s cells in the complex with MHC molecules (“unique recipient immunopeptides,” URiPs). URiPs represent potential MiHAs. More accurately, there is a third step that comprises prediction of URiP immunogenicity, i.e., the ability to induce a specific immune response. However, at this moment it is not clear which factors can influence the magnitude of an immune reaction. For that reason we restricted ourselves to the first two steps. In the first part of this paper, we described the features of 9-mer peptidome unique for the recipient, and further we described the features of *in silico* predicted unique immunopeptidome presented by the common class I HLA alleles, namely, HLA-A*01:01, HLA-A*02:01, HLA-A*03:01, HLA-A*11:01, HLA-A*24:02, HLA-B*07:02, HLA-B*08:01, HLA-B*15:01, HLA-B*35:01, HLA-B*44:02, and HLA-B*57:01. Note that MHC binding prediction for all samples was performed for the same set of alleles independent of actual HLA-genotypes of the individuals. This was done for the reason that the dataset contained only four HLA-matched pairs. However, we showed that the number of predicted binders is very consistent between samples, and that overall genomic disparity between samples did not depend on HLA-allele matching.

**Figure 1:**
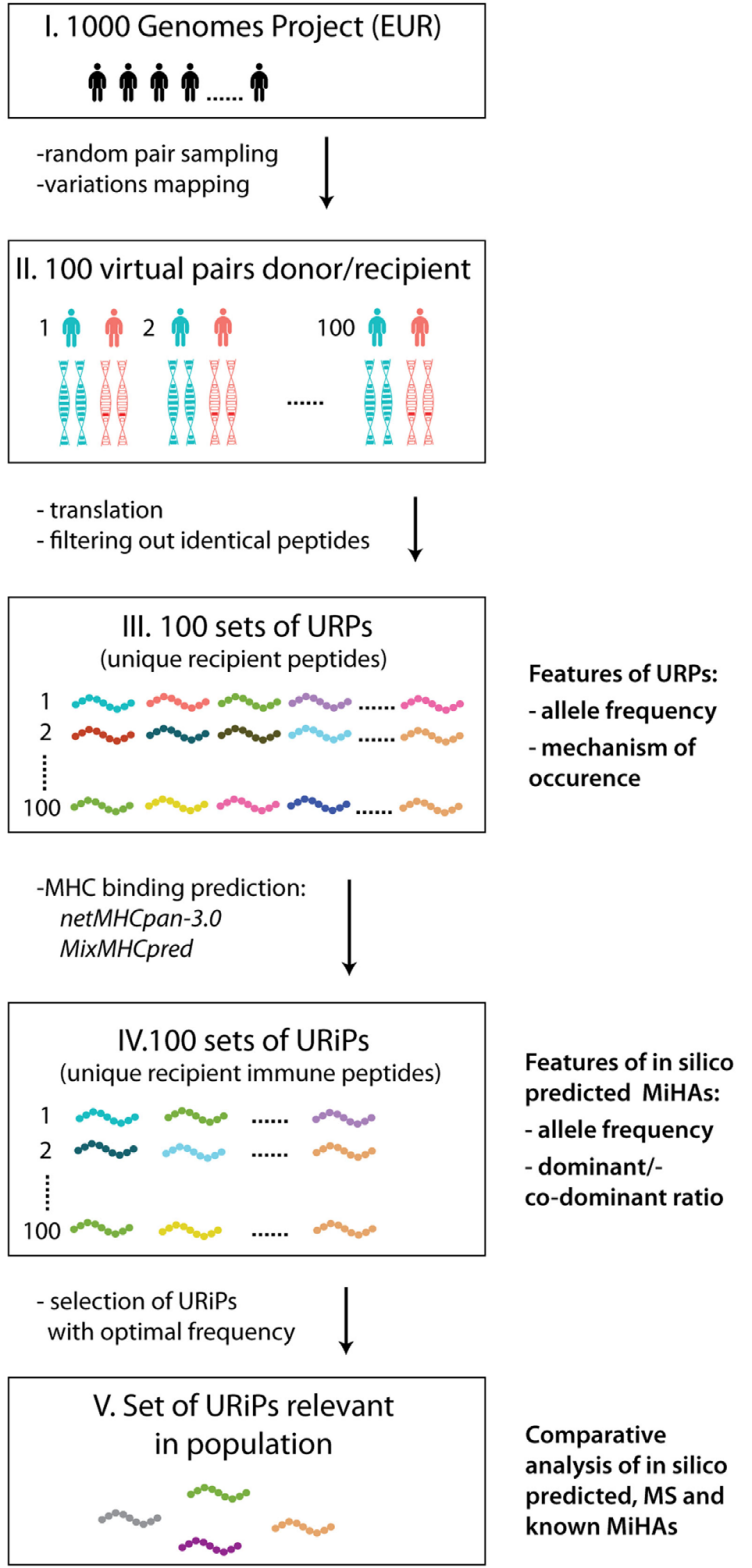
The workflow of the study. The main steps of the study are schematically depicted: 100 sample pairs of European origin were randomly selected from the 1000 Genomes Project as virtual “donors” and “recipients” (I). The variation data were used to obtain individual proteomes (II). For each pair peptides unique for recipient (URPs) were identified (III). For all URPs MHC binding to the most frequent HLA alleles was predicted with two MHC-binding programs, non-binding peptides were filtered out. The peptides obtained at this stage are designated as unique recipient immune peptides (URiPs) (IV). On the last stage only therapeutically relevant peptides, i.e., with high probability of mismatch, were left (V). On the right side of the figure the analysis applied to the data on each step is shown: (1) for URPs: analysis of encoding allele frequency and the type of encoding polymorphisms, (2) for URiPs: analysis of encoding allele frequency and co-dominant/dominant ratio, and (3) comparative analysis of URiPs, mass spectrometry data, and known minor histocompatibility antigens.

Our workflow of MiHA prediction is depicted at the Figure 1. First, 100 pairs of samples were randomly selected from 503 available unrelated samples of European origin (populations IBS, FIN, TSI, GBR, and CEU). Each sample was allowed only once, i.e., 200 distinct samples were randomly assigned to 100 pairs. The IDs of selected samples are listed in Table S1 in Supplementary Material. For URPs identification in each pair, individual sample proteomes were obtained for a virtual “donor” and “recipient” by mapping the variation data on the reference genome and subsequent translation of protein-coding transcripts (in-house made script). The individual proteomes were further cut into 9-mer peptidomes. The set of unique recipient peptides was obtained by subtracting the peptidome of the “donor” from the peptidome of the “recipient”. Each particular URP was assigned to the respective encoding allelic variant(s). The examples of possible allelic combinations leading to URP formation are schematically depicted in Figure S1 in Supplementary Material. We distinguished several main classes of URPs by their origin; URPs were caused by one or several adjacent nsSNPs, by in-frame insertions or deletions, by frameshift mutations in recipient, homologous frameshift or nonsense mutation in the donor. In rare cases, URPs were caused by a combination of polymorphisms.

The obtained URP sets were then subjected to the MHC-binding prediction for 11 common HLA alleles. URiPs with the optimal frequency of occurrence were defined as suitable for the therapeutic usage.

### Prediction of Peptide-MHC Class I Affinity

Two algorithms were used to predict peptide affinity to MHC proteins: NetMHCpan-3.0 (31) with the default thresholds for strong (rank < 0.5) and weak (rank < 2) binders; and MixMHC which was recently developed based on the large MS immunopeptidome dataset (32). The *p*-value thresholds for strong (SB) and weak binders (WB) for MixMHC were chosen as 0.005 and 0.02, respectively. Both rank and *p*-value thresholds represent the fraction of positive predictions in the set of peptides randomly selected from the human genome. I.e., all URPs predicted with the 0.005 MixMHC threshold are among the top 0.5% binding peptides from human genome. The rank thresholds 0.5 and 2 of NetMHCpan-3.0 correspond to *p*-value thresholds 0.005 and 0.2 of MixMHC, respectively. Such percentile-based thresholds demonstrated better performance in comparison with the affinity-based thresholds (31). To strengthen the reliability of predictions and reduce the number of candidates we also considered the overlapping predictions, i.e., peptides predicted as SB or WB by both programs.

### Datasets of MS Analysis of Immunopeptidome

Mass spectrometry dataset used in this paper was collected following Bassani-Sternberg et al. (32) from publicly available MS datasets (33–39). The HLA restriction assignment was done *de novo* with MixMHC program using the HLA-genotyping data for the samples specified in the studies. For the data from Abelin et al. (39) there was only one HLA per sample, thus, the prediction of HLA restriction was not performed. The full MS dataset used in the paper can be found in Table S4 in Supplementary Material.

## Results

### Peptidome Unique for Recipient

We analyzed the genomic variation data for 100 pairs of unrelated individuals belonging to the European populations, arbitrarily defining one of them as a virtual “recipient” and the other as a virtual “donor” (Figure 1). For each pair, we took 34,077 protein-coding transcripts, confirmed by RefSeq, into consideration (see Materials and Methods). First, we calculated the number of allelic variants that were unique to the recipient genome, which was 11 243 ± 255 (Table S1 in Supplementary Material). A fraction of these allelic variants changes the amino acid sequence of the encoded protein and thus, gives rise to the new peptides, which are potentially presented in complex with MHC. We further focused on the nature and number of the peptides that were unique to the recipient.

**Figure 2:**
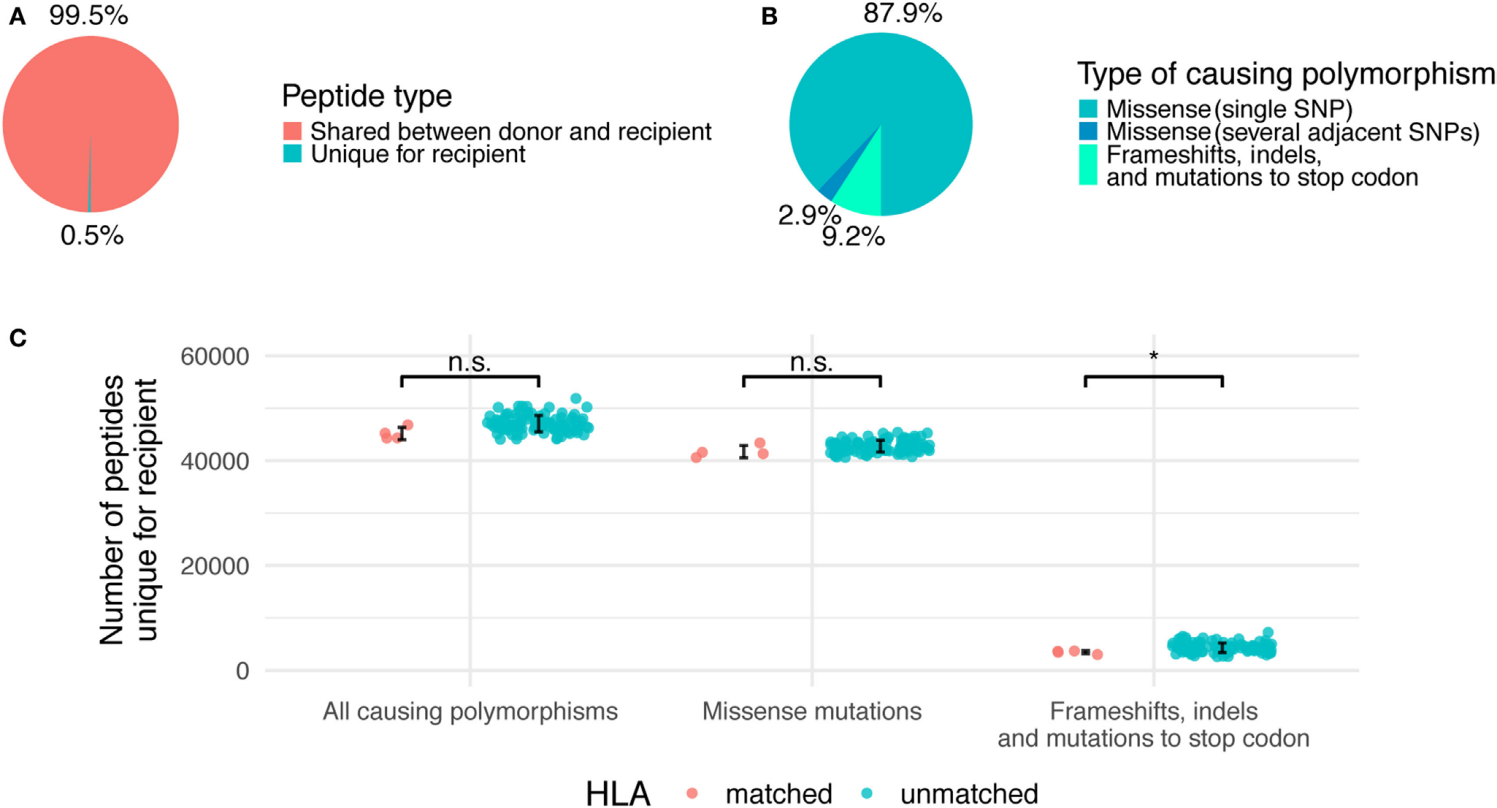
Relative contribution of different genetic polymorphisms to unique recipient peptidome. **(A)** The fraction of total recipient’s peptidome occupied by unique recipient peptides (URPs). **(B)** The share of URPs encoded by different polymorphisms. **(C)** Absolute numbers of URPs in all analyzed pairs, HLA-matched and unmatched pairs are shown separately. Error bars mark one SD from the mean. Significance of two-tailed *t*-test for comparison of the two groups is shown as follows: *p* < 0.05 (*), n.s, non significant.

Most of the peptides presented in the MHC I are 9 amino acids long (the exact percentage can vary between distinct HLA alleles) (35). In line with this and for simplification, we restricted our analysis to 9-mer peptides. To obtain all the peptides unique to recipients, individual peptidomes of the donors were subtracted *in silico* from the peptidomes of the recipients in each pair. As a result, the average number of 9-mer unique recipient peptides (URPs) per pair was found to be 47,057 ± 1,566, which occupied 0.47 ± 0.02% of the whole recipient 9-mer peptidome (Table S1 in Supplementary Material; Figures 2A,C). This suggested that the ratio of URiPs to all peptides presented on the surface of the recipient cell was approximately 1:200, as we did not expect any bias toward variant or invariant peptides in the antigen presentation machinery. Consequently, low estimation of MiHA numbers in an unrelated donor-recipient pair can be defined around 250, as individual immunopeptidome diversity is at least 50,000 according to estimations from MS experiments (33).

The majority of the obtained URPs (88 ± 2%) were caused by nsSNPs encoding a single amino acid substitution in the donor–recipient pair of homologous peptides, while another 3 ± 0.2% were caused by several adjacent SNPs that resulted in 1 or more amino acid substitutions. This is in line with the fact that most of the known MiHAs are associated with a single nsSNP (11). The remaining 9 ± 1% of URPs were caused by frameshifts and indels in donor or recipient genomes, or by homozygous nonsense mutations in the donor (Figure 2B). Among non-nsSNP peptides, 11 ± 3% were due to indel in the recipient or donor, 19 ± 7% due to frameshift in the recipient, 40 ± 13% due to homozygous frameshift in the donor, 28 ± 11% due to homozygous nonsense mutation in the donor, and 1 ± 1% due to the combination of several reasons. The detailed counts of peptides per polymorphism type in the studied pairs are given in Table S2 in Supplementary Material. As expected, the average number of variant peptides per polymorphism was 9 for missense SNPs and small in-frame indels, whereas the distribution was much broader for frameshift and nonsense mutations (Figure S2 in Supplementary Material). Some of the polymorphisms can result in thousands of URPs when found in the homozygous state in the donor. In this situation, the source of the mismatched peptides is unaltered recipient protein, whereas only truncated protein is present in the donor. Notably, truncated proteins are a relatively common phenomenon. An estimated 20 complete loss-of-function proteins are present in the homozygous state per individual; most of them are caused by frameshifts, which lead to premature protein truncation (40).

Since URP-coding SNPs are evenly distributed across the genome, most of them are not in linkage disequilibrium with HLA alleles. Thus, we demonstrated that the number of URPs in HLA-matched pairs do not significantly differ from that in random pairs, i.e., obtained estimations could be extrapolated to actual HLA-matched unrelated allo-HSCTs (Figure 2C).

Altogether, 672,913 distinct URPs were found in all 100 considered pairs, thereby showing that most URPs are repeated among pairs (see below). The probability of finding a particular URP in a pair depends on the frequency of the corresponding encoding allele (referred below as allele frequency, *f*) in the population. It is described by the following formula:

$$P_{\text{mm}}=f\times {\left(1-f\right)}^{2}\times \left(2-f\right).$$

**Figure 3:**
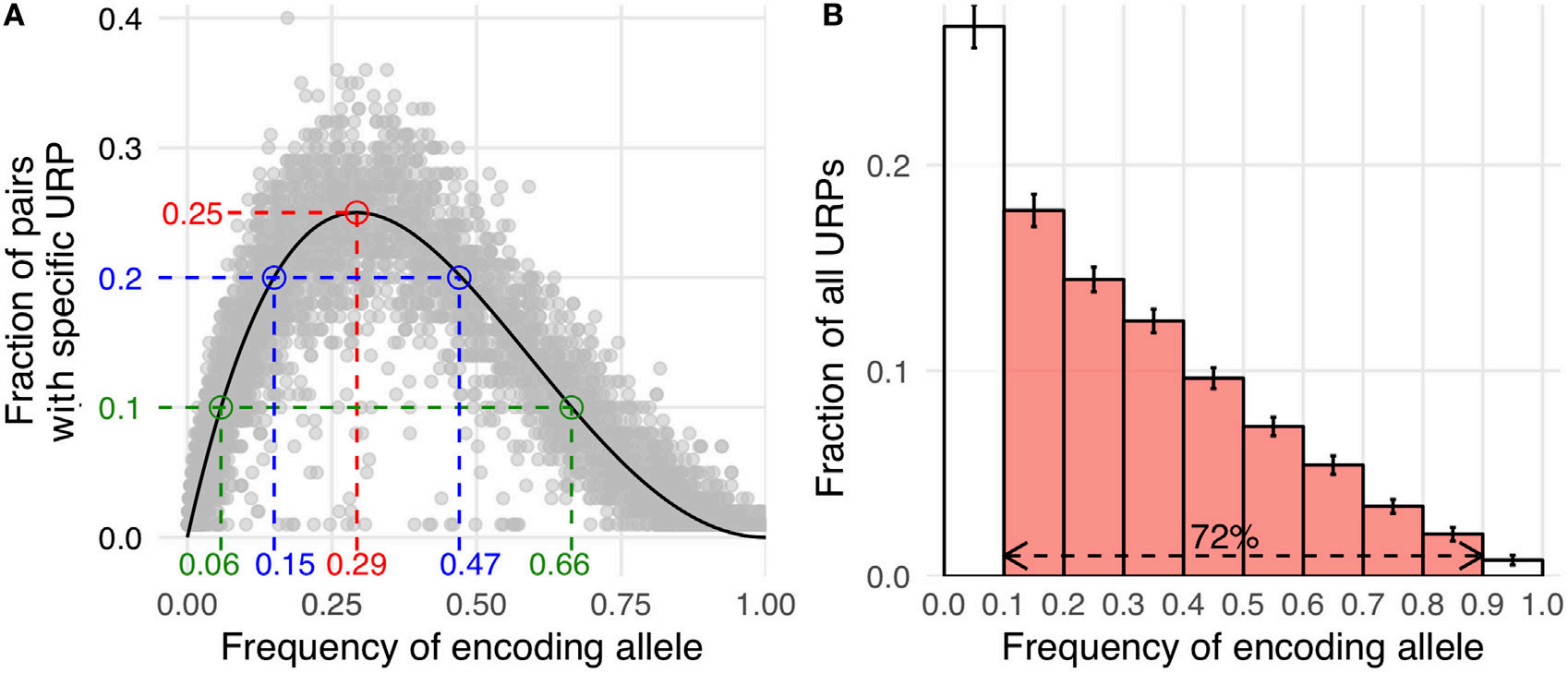
Correlation of allele frequency and unique recipient peptides (URP) occurrence. **(A)** Allele frequency of encoding polymorphism is plotted against the fraction of pairs in which this particular URP was found (random selection of 10,000 URPs is shown). Theoretical curve calculated by the formula provided in the text is shown in black. Red dashed line indicates the peak at which Pmm (probability of minor histocompatibility antigen mismatch) reaches 25%. Blue and green lines delineate allele frequency range yielding Pmm ≥ 20 and 10%, respectively. **(B)** The distribution of nonsynonymous polymorphism-encoded URPs by the frequency of encoding allele. The data were averaged among all considered pairs, error bars show one SD. The red fill indicates the fraction of URPs encoded by the alleles relatively frequent in the population (0.1 < MAF < 0.9).

**Figure 4:**
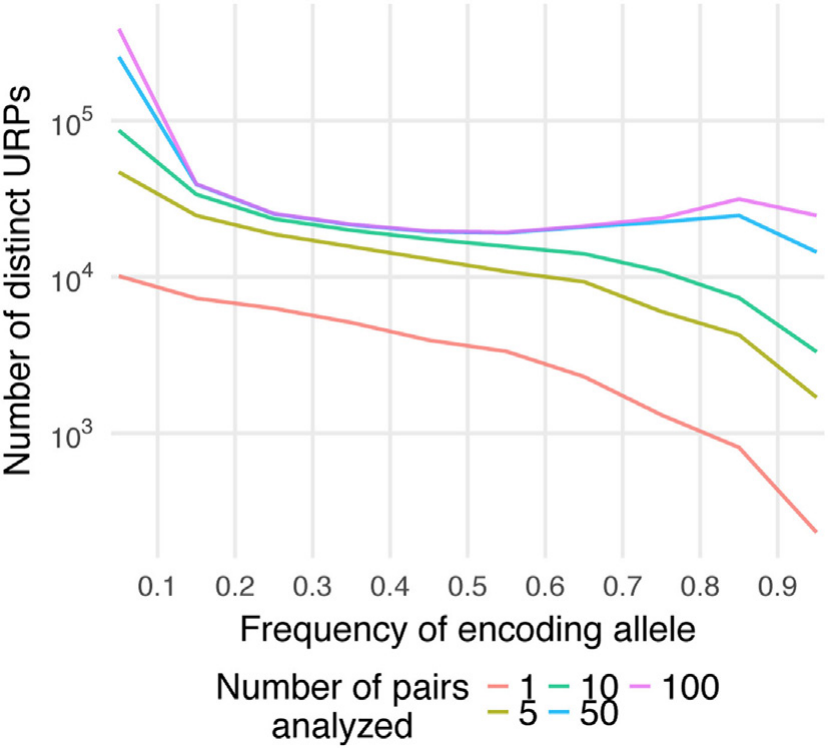
Saturation of the number of distinct unique recipient peptides (URPs). The encoding allele frequency (*f*) distribution of distinct URPs is plotted separately for different number of analyzed pairs (1–100). Note that *Y*-axis is in logarithmic scale.

Here, P_mm_ is the probability of MiHA mismatch and *f* is the frequency of the MiHA-encoding allele in the population. It is a product of the probability that the MiHA-encoding allele is absent in the donor and the probability that it is present in the recipient (for derivation of the formula see Supplementary Text in Supplementary Material). The same formula, as a function of the donor allele frequency, was obtained earlier as well (41). This formula approximates well with our data (Figure 3A). It implies that the probability of a specific URP-mismatch non-linearly depends on the *f* of the corresponding polymorphism, which favors polymorphisms whose alternative (not MiHA-encoding) allelic variant is more frequent in the population. Maximum P_mm_ (0.25) is achieved at $f=1-\frac{1}{\sqrt{2}}$ (0.293). The optimal window to search for therapeutically relevant MiHAs (P_mm_ > 0.1) would be in the *f* range of 0.06–0.65 for URP-coding alleles, while the most relevant MiHAs (P_mm_ > 0.2) would be found in *f* range of 0.15–0.47 (blue and green dashed lines, respectively in Figure 3A). We then analyzed the distribution of nsSNP-encoded URPs in a pair with respect to the frequency of the encoding allele. We found that 7.9 ± 0.9% of URPs were coded by very low-frequency alleles (*f* < 0.01), 18.8 ± 0.9% were coded by low-frequency alleles (0.01 < *f* < 0.1), and 72.5 ± 1.1% were coded by common alleles, with an *f* range of 0.1–0.9. Only 0.8 ± 0.2% of URPs were encoded by alleles with *f* > 0.9 (Figure 3B, the data for each pair can be found in Table S2 in Supplementary Material). The minor contribution of polymorphisms encoded by frequent alleles to overall URP space is explained by the diminishing probability for a donor to be homozygous by the alternative allele. This is in line with previous experimental data (12), where no specific immune target was assigned to 30% of the alloreactive clones most probably because the frequency of the corresponding polymorphism was lower than the detection threshold. The URP saturation curve (increase in the number of unique URPs with the addition of new pairs to the analysis) is also highly *f*-dependent (Figure 4). When taking 100 pairs of individuals into consideration, we obtained almost all (over 99.99%) relevant URPs (P_mm_ > 0.1).

Using HLA-matched siblings as donors is considered to be the gold standard for allo-HSCT, since these kinds of transplantations are associated with lower incidences of GvHD than transplantations from unrelated HLA-matched donors. Sibling donors have a 25% probability of sharing both HLA haplotypes and they are expected to share more allelic variants of MiHAs with the recipient than unrelated donors. Therefore, we also studied sibling pairs available on the 1000 Genomes project data (see Materials and Methods). The observed number of URPs were approximately 1.8-folds less for siblings than for unrelated pairs (Figure S3 and Table S1 in Supplementary Material), which closely matches previously obtained results (42, 43). P_mm_ for sibling pairs was calculated using the following formula:

$$P_{{\text{mm}}_{\text{Sib}}}={\left(1-f\right)}^{2}\times f\times \left(4-f\right)/4,$$

where *f* is the frequency of the MiHA-encoding allele in the population (for derivation of the formula see Supplementary Text in Supplementary Material). The maximum P_mm_ for sibling pairs is 0.136, which is achieved at $f=7-\frac{\sqrt{33}}{4}$ (0.314) (Figure S4 in Supplementary Material).

### Features of *In Silico* Predicted Potential MiHAs

Next, we studied URPs that were *in silico* predicted to bind to MHC proteins (see Materials and Methods), henceforth addressed as “unique recipient immunopeptides” (URiPs). We utilized two independent algorithms: widely used netMHCpan-3.0, which implemented neural networks (31), and recently released PSSM matrices, which were trained on an extensive experimental dataset (32). Both programs were used to predict strong and weak MHC-binding URiPs.

First, we analyzed the URiPs in the P_mm_ > 0.1 region (where URPs were saturated, allowing us to detect nearly all the URPs in the population in this region). The number of strong binders (SB) predicted by netMHCpan-3.0 (rank < 0.5, see Materials and Methods) ranged from 750 to 1,758 for HLA-A*01:01 and HLA-B*07:02, respectively, whereas the number of weak binders (WB) (rank < 2) from 3,097 to 6,523 for HLA-A*01:01 and HLA-B*35:01, respectively (note that WB by definition included SB). Analysis with the PSSM prediction algorithm using the same *p*-value thresholds resulted in less variable estimations among the HLA alleles. Strong binders ranged from 633 to 888 for HLA-B*44:02 and HLA-B*07:02, respectively, whereas the number of weak binders ranged from 2,713 to 3,634 for HLA-A*01:01 and HLA-B*07:02, respectively (Table 1). However, the predictions of the two programs only partially overlapped. The number of overlapping strong binders ranged from 277 to 657 for HLA-B*15:01 and HLA-B*07:02, respectively, whereas the number of overlapping weak binders ranged from 1,511 to 2,519 for HLA-B*15:01 and HLA-B*07:02, respectively (Table 1). To increase the odds of actual MHC-binding by the peptide and to decrease the number of MiHA candidates, we used stringent criteria and only considered peptides that were predicted as strong or weak by both algorithms in the downstream analysis. The overlapping strong binders in the P_mm_ > 0.1 region are listed in Table S3 in Supplementary Material as potential MiHAs.

**Table 1:** Number of predicted unique recipient immunopeptides (URiPs) and mass spectrometry (MS) peptides in the region where P_mm_ > 10 and 20%.

HLA allele	NetMHCpan WB	NetMHCpan SB	MixMHC WB	MixMHC SB	Overlap WB	Overlap SB	MS peptides

Region of P_mm_ > 10%							
A*01:01	3,097	750	2,713	634	1,684	390	9
A*02:01	4,161	1,400	3,032	717	1,782	451	20
A*03:01	3,167	923	2,771	647	1,864	404	23
A*11:01	4,002	1,268	2,969	693	1,936	473	2
A*24:02	3,419	984	2,728	638	1,641	404	4
B*07:02	5,372	1,758	3,634	888	2,519	657	40
B*08:01	4,779	1,395	2,989	691	1,838	377	15
B*15:01	3,822	1,076	2,848	681	1,511	277	7
B*35:01	6,523	1,724	2,953	706	2,133	488	16
B*44:02	3,198	894	2,768	633	1,623	373	4
B*57:01	3,291	941	2,907	827	1,797	515	9

**Region of P_mm_ > 20%**							

A*01:01	1,495	357	1,325	293	801	185	2
A*02:01	2,025	699	1,464	361	875	226	9
A*03:01	1,497	449	1,306	330	874	200	13
A*11:01	1,864	622	1,411	313	903	215	1
A*24:02	1,635	482	1,330	304	795	192	1
B*07:02	2,571	845	1,740	420	1,238	322	23
B*08:01	2,289	696	1,466	323	896	183	6
B*15:01	1,890	519	1,405	342	746	141	6
B*35:01	3,135	846	1,422	344	1027	238	12
B*44:02	1,540	430	1,338	323	783	193	1
B*57:01	1,567	444	1,387	1387	856	254	5

**Figure 5:**
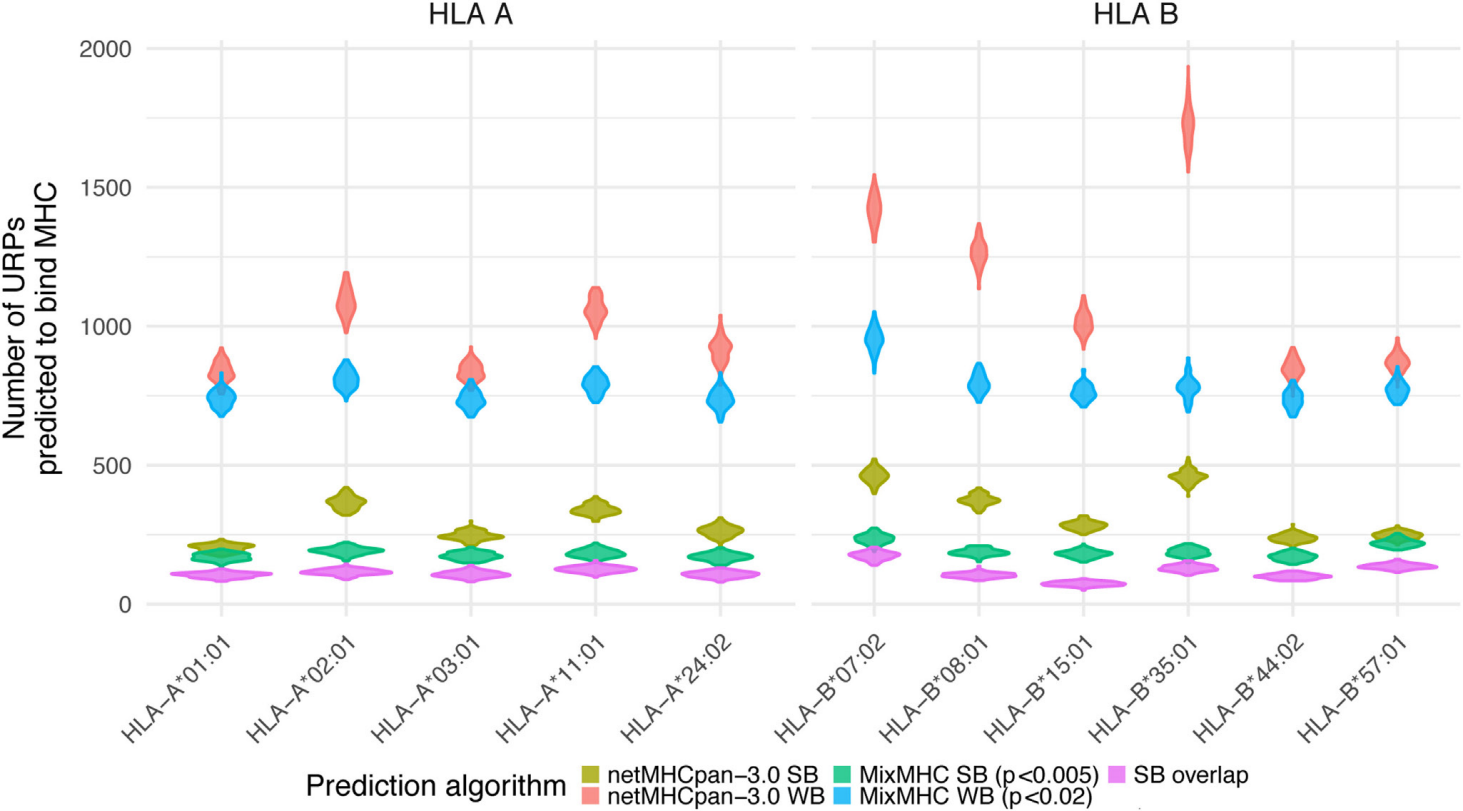
Unique recipient peptides (URPs) predicted to bind MHC. The number of URPs predicted to bind MHC is shown for 11 most frequent HLA alleles, variance among pairs is depicted with violin density plots. The results of different prediction programs used with different prediction thresholds are shown with color. SB – strong binders; WB – weak binders; SB overlap – strong binders predicted by two independent algorithms.

The distribution of URiPs among the studied pairs showed that the variation in URiPs numbers was dictated by the HLA allele rather than genetic variation (Figure 5). Moreover, the number of predicted URiPs per HLA corresponded to their binding promiscuity, which was calculated using a random sample from the genome (Figure S5 in Supplementary Material). URiP distribution with respect to the encoding allele frequency was not significantly different from the URP distribution (Figure S6 in Supplementary Material). Thus, we confirmed that MHC-binding did not introduce any bias into the *f* profile and that the above described dependency between P_mm_ and *f* was also valid for URiPs.

### Intersection of URPs With MS Data and Reported MiHAs

Mass spectrometry of peptides eluted from pMHC complexes is extensively used to examine immunopeptidomes and is believed to be significantly more accurate than *in silico* prediction of antigen presentation as a strategy to search for novel MiHAs and neoantigens (19, 36, 44, 45). To study the extent to which MS data intersected with predicted URPs and URiPs, we accumulated a large set of published MS-identified peptides described in the literature (32, 33, 39). The dataset included 89,324 distinct 9-amino acid long sequences, of which 74,007 were assigned to either HLA-A or HLA-B alleles by the MixMHC program. We focused our study on this dataset (Table S4 in Supplementary Material). Next, we analyzed the intersection of this dataset with the URPs and URiPs reported here.

**Figure 6:**
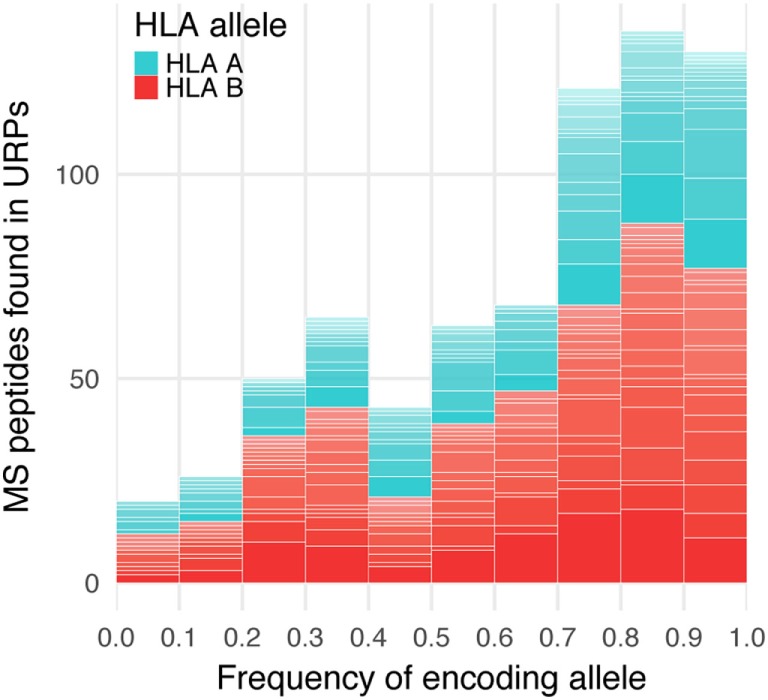
Encoding allele frequency of mass spectrometry (MS)-verified unique recipient peptides (URPs). The peptides obtained at the intersection of URPs and MS datasets were grouped according to the frequency of URP-encoding allele. The columns are divided at segments corresponding to the assigned HLA-restricting allele.

The vast majority (99.2%) of the 9-mer peptides detected by MS did not intersect with the URP set obtained in our experiments, which suggested that they were encoded by the invariant loci. 565 distinct URPs found in MS dataset represent naturally processed and presented peptides. These peptides are potential MiHAs that require immunogenicity validation (they are listed in Table S4 in Supplementary Material). Most of the variant peptides in the MS dataset were predicted to be MHC-binders *via* the stringent approach that utilized two independent algorithms implemented here. On average, 77 ± 17 and 48 ± 19% of the variant MS peptides were reported to be weak and strong binders, respectively among considered HLA alleles, whereas this share was 84 and 51%, respectively for the most frequent allele (HLA-A*02:01) in European population (Table 2). Strikingly, when we analyzed the *f* distribution of the URPs found in the MS data, we found that it was significantly skewed toward a higher *f*, i.e., toward URPs with non-optimal P_mm_ (Figure 6). It is unlikely that MS exhibits some intrinsic bias: thus, the skewness of the data toward peptides encoded by higher frequency alleles may be explained by two factors. It could either be caused by the usage (at least in some experiments) of reference genome data for peptide mapping or by the insufficient number of experiments. The rare allelic variants certainly require more samples to be detected.

**Table 2:** Fraction of peptides unique for recipient (URPs) from mass spectrometry (MS) data predicted to bind MHC.

HLA allele	Number of URPs in MS data	Of them in URiP weak binders (WB)	Of them in URiP strong binders (SB)	Fraction from MS URPs (WB)	Fraction from MS URPs (SB)
A*01:01	14	6	4	0.43	0.29
A*02:01	45	38	23	0.84	0.51
A*03:01	48	34	25	0.71	0.52
A*11:01	3	3	1	1.00	0.33
A*24:02	13	11	9	0.85	0.69
B*07:02	88	69	44	0.78	0.50
B*08:01	29	27	19	0.93	0.66
B*15:01	11	6	2	0.55	0.18
B*35:01	17	12	5	0.71	0.29
B*44:02	24	22	19	0.92	0.79
B*57:01	20	15	11	0.75	0.55

			**Mean**	0.77	0.48

			**SD**	0.17	0.19

Minor histocompatibility antigens with high P_mm_ (>0.2) represent prime therapeutic targets as they are mismatched in a substantial number of donor–patient pairs. However, the available MS data contain very few of them due to the above-described bias. Even for alleles with the highest number of MS-reported peptides (HLA-B*07:02, HLA-A*03:01, and HLA-A*02:01), only a few peptides with P_mm_ > 0.2 exist (23, 13, and 9, respectively), whereas the *in silico*-predicted URiP dataset contains 322, 200, and 226 strong MHC binders, respectively. This suggests that a substantial number of high therapeutic potential MiHAs may be still undiscovered (Table 1).

Another evidence of MS data deficiency in the high P_mm_ region was that most of the known MiHAs were not detected in the MS experiments. Out of 35 previously reported 9-mer MiHA peptides [reviewed in Ref. (11)], only 6 were present in the studied MS dataset, whereas 32 of them were present in the URP set (three peptides were not found because they were caused by entire gene deletion or were transcribed from an alternative reading frame and thus, were beyond the scope of our approach). Out of 32 detected URPs, 27 (84%) weak- and 20 (62.5%) strong-binding URiPs were predicted (Table S5 in Supplementary Material). This suggests that around 60% of still undiscovered MiHAs would be contained in the strong binder set predicted by the two algorithms. Given that the number of such URiPs for each allele is not prohibitively high, *in silico* reverse immunology approach followed by *in vitro* immunogenicity testing can be feasibly used to discover them.

### Co-Dominant/Dominant MiHA Ratio

Most MiHAs are encoded by biallelic nsSNPs, and they fall into 1 of 2 principal groups depending on whether both or just 1 of the allelic variants encode peptides presented in the immunopeptidome. A MiHA allele is termed dominant when its peptide is exclusively presented in MHC; the counterpart allele coding non-presented peptide is accordingly designated as recessive. When both alternative alleles encode peptides presented in the immunopeptidome, they are referred to as co-dominant (46). Although in-depth knowledge is lacking as to how these two groups of MiHAs differ from each other with respect to their immunogenicity or other features, the presence or absence of counterpart peptides can theoretically influence allogeneic immune responses to MiHA targets. From another perspective, the co-dominant to dominant MiHA ratio represents a fundamental feature of the antigen presentation machinery; in essence, it describes the extent to which a single amino acid substitution affects proteasomal degradation and MHC binding. We aimed to calculate this value in our dataset and compare it to the co-dominant/dominant ratio of reported MiHAs and MS-detected peptides.

**Figure 7:**
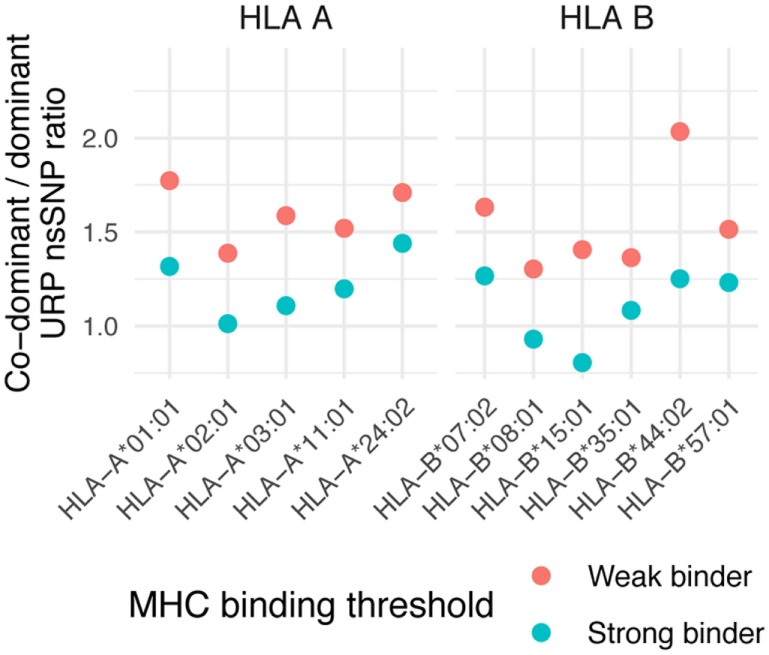
Co-dominant/dominant ratio of unique recipient immunopeptide (URiP)-encoding nonsynonymous polymorphisms (nsSNPs). The number of URiP-encoding nsSNPs predicted to be co-dominant (i.e., both allelic variants give rise to peptides predicted to bind specific MHC) divided by the number of dominant nsSNPs. Only the concordant predictions between programs (shown as “overlap” at Figure 5) were used for both strong and weak binding thresholds.

The co-dominant/dominant ratio for all URiP-encoding nsSNPs in our data is 1.15 ± 0.18 for strong and 1.57 ± 0.21 for weak binders (mean value among considered HLA alleles ± SD, Figure 7). To isolate the possible effect of differential proteasome degradation we took advantage of a recently published finding (39) which reported that only mutations to arginine and lysine contained in a peptide significantly increased the probability of internal proteasomal cleavage. The exclusion of all peptides containing arginine or lysine from the analysis yielded a more balanced ratio of polymorphisms representing strong (0.8 ± 0.14) and weak (1.03 ± 0.13) binders. Thus, our analysis suggests that the number of co-dominant MiHAs should be comparable to that of dominant MiHAs. Strikingly, the dominant variant peptides are much more abundant in MS data, resulting in a reversed co-dominant/dominant ratio. Only 28 (of 489) nsSNP demonstrated both variants (i.e., confirmed co-dominant *via* MS) in the dataset used in this study, resulting in a co-dominant/dominant ratio of 0.06 (Table S4 in Supplementary Material). The same tendency was reported in other MS studies (19, 46). Among 30 known 9-mer MiHA-encoding nsSNP polymorphisms, only 2 were reported as co-dominant, resulting in a ratio of 0.07 (Table S5 in Supplementary Material). For known MiHAs, the independent discovery of two variants of the same MiHA is very unlikely as only a small number of MiHAs have been discovered and the systematic investigation of counterpart peptide immunogenicity is usually not performed. Recent quantitative MS experiments, focused on specific MiHAs rather than the whole immunopeptidome, confirmed that some MiHAs, previously thought to be dominant, were actually co-dominant (47). If the MiHA co-dominant/dominant ratio suggested by *in silico* analysis is valid, approximately half of the MiHAs would be categorized as co-dominant and the number of targetable antigens would substantially increase.

## Discussion

Many sophisticated constructs were developed recently to facilitate T-cell immunotherapy. For instance, T-cell transduction with transgenic TCRs (10) and chimeric antigen receptors (48) created antigen-specific T cells *in vitro*, while bispecific T-cell engaging antibodies (49, 50) and ImmTACs (51) changed the specificity of the cell *in vivo* effectively redirecting any T cell to the desired antigen. However, in the end, immunotherapy is only as good as its antigenic target. Tumors of the hematopoietic system bear relatively low mutational load (52); thus, the use of neoantigens is restricted. Some tumors express CD19 and are targetable by anti-CD19 CAR T cells; however, acute myeloid leukemia, the most common hematopoietic malignancy in adults, lacks this marker. MiHAs represent an appealing alternative target for immunotherapy following allo-HSCT. Only a small part of them are hematopoietically restricted; a feature necessary to avoid potentially detrimental off-tumor targeting. Another limitation is that the maximum frequency of MiHA mismatch is only 25%, and restriction by a particular HLA allele lowers the number of eligible patients even more. To use MiHAs as therapeutic targets on a large scale, a reasonably sized panel of MiHAs is needed. This highlights the demand for the discovery of novel hematopoietic MiHAs. Since tumors are prone to immune escape by downregulating HLA expression or *via* homologous recombination in the HLA locus that leads to the loss of HLA heterozygosity (53), the simultaneous use of several MiHAs, restricted by different HLA, is ideal. Currently, a total of around 70 MiHAs with confirmed immunogenicity are known (a few of them are suggested to be hematopoetically restricted). The number of undiscovered MiHAs remains unknown. This knowledge will determine the applicability and potential efficacy of anti-MiHA immunotherapy. Here, we attempted to draw attention to the importance of systematic MiHA discovery.

Although *in silico* approaches cannot provide the exact set of immunogenic targets, we focused on the descriptive features of the immunopeptidome that are unlikely to be influenced by the biases of the antigen presentation machinery. Our study revealed the following features. Only 0.5% of the immunopeptidome targets in the patient (also referred to as URPs) were absent in the donor, i.e., they were unique to the recipient. Most of the variant peptides (90%) were caused by nsSNP polymorphisms, which was in line with the existing experimental data. We showed that most of URPs and potential MiHAs in a given donor/recipient pair are caused by polymorphisms common in the population (MAF > 0.1).

The pioneering work of Granados et al. (19) demonstrated that MiHAs could be detected using proteogenomic approach. More importantly, all the tested predicted peptides appeared to elicit an immune response. However, evidence also suggests that bulk MS studies could significantly underestimate the size of the immunopeptidome. For example, Kumari et al. (22) and Gubin et al. (21) showed that T cells could recognize epitopes that were undetected in bulk MS experiments. The immunopurification step in immunopeptidome analysis was also shown to capture around 5% of all peptides, essentially detecting only the most common epitopes (54). Upon analyzing allele frequency distribution of MS-predicted MiHAs, we found that most of MS peptides were found in the region of frequent immunogenic alleles, while only a few peptides were present in the region optimal for therapeutic usage (around allele frequency 0.3). Although MS experiments are highly consistent in immunopeptidome and MiHA recovery (19, 55), it is still to be determined whether they systematically underestimate the number of immunogenic MiHAs or there is indeed a limited number of them. Indirect evidence suggesting that MiHA numbers might be underestimated during MS appears *via* the reversed ratio of co-dominant and dominant MiHAs observed in the MS data when compared to *in silico* predictions.

We have provided a list of the best *in silico*-predicted 9-mer MiHAs for the most frequent MHC alleles in the ideal P_mm_ range (over 10%). Analysis of the known MiHAs suggested that a list obtained with selected prediction thresholds should contain 50% of all MiHAs in this region. The relatively small number of peptides (from 100 to 200 peptides per each allele), which is still about 10 times more than is suggested by the MS data, provides the possibility of systematically checking their immunogenicity. The feasibility of reverse immunology approach followed by immunogenicity verification was shown for neoantigens (25). In our opinion, this approach will facilitate the systematic examination of the MiHA landscape and yield therapeutically relevant targets.

In the present work, we restricted analysis to 9-mer peptides translated in normal reading frames from protein-coding transcripts. However, it is known that T-cell can recognize 10- or 11-mer peptides, moreover T-cell targets can be translated from protein-coding transcripts in alternative reading frames or from alternative transcripts (11). As such, it is possible that there are additional sets of URPs that were not considered here.

## Data Availability Statement

The datasets used in this study can be found in Supplementary Materials for this article.

## Author Contributions

NB, DM, and GE designed the study. NB performed the analysis. NB and GE wrote the manuscript. All authors read and reviewed the manuscript.

## Conflict of Interest Statement

The authors declare that the research was conducted in the absence of any commercial or financial relationships that could be construed as a potential conflict of interest.
