# Supplementary material for: In Silico Analysis of the Minor Histocompatibility Antigen Landscape Based on the 1000 Genomes Project
Source: Front Immunol. 2018 Aug 16;9:1819. doi: 10.3389/fimmu.2018.01819 (PMC6105694; doi:10.3389/fimmu.2018.01819)
Supplement: Supplementary file 6 [file data_sheet_1.PDF]

Supplementary Material

In Silico Analysis of the Minor Histocompatibility Antigen Landscape Based on 1000 Genomes Project

Nadia A. Bykova [1*], Dmitry B. Malko [1], Grigory A. Efimov[1*]

[1]Laboratory of Transplantation Immunology, National Research Center for Hematology, Moscow, Russia

*** Correspondence:**

Nadia Bykova, 4noodle@gmail.com
Grigory Efimov , PhD, MD efimov.g@blood.ru

1 Supplementary Figures

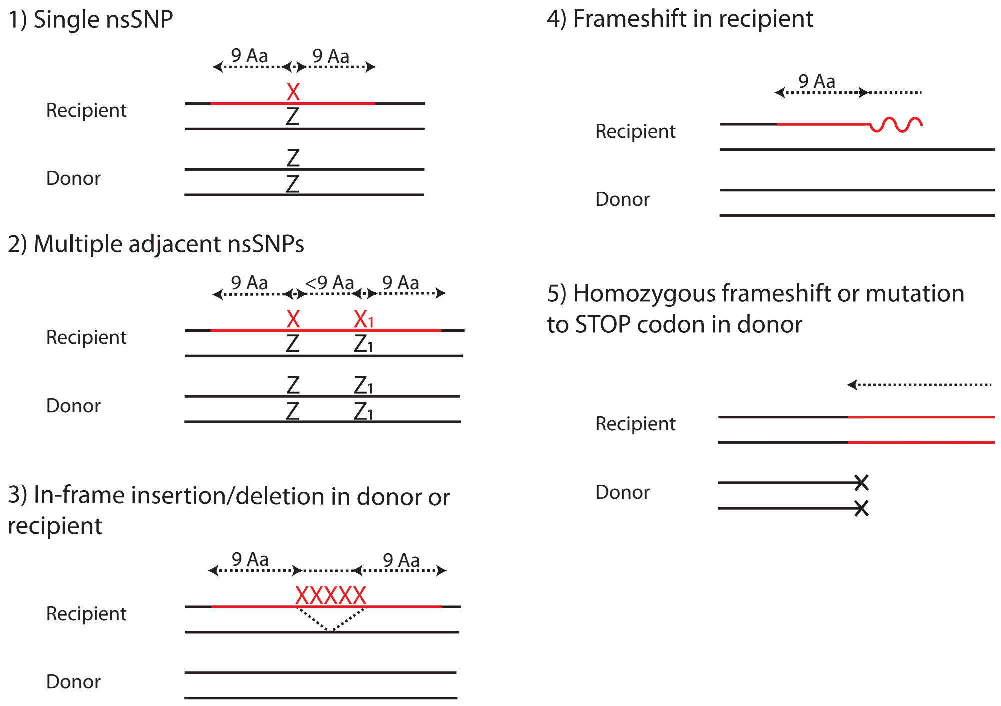

Supplementary Figure 1. The mechanisms of URP (unique recipient peptides) occurrence. Five situations considered in this paper that can lead to the occurrence of URPs are schematically shown: 1) single nsSNP, 2) several adjacent nsSNPs, 3) in-frame insertion or deletion, 4) frameshift in recipient, and 5) homozygous frameshift or mutation to STOP codon in donor. The region of recipient's protein that gives rise to URPs is shown in red.

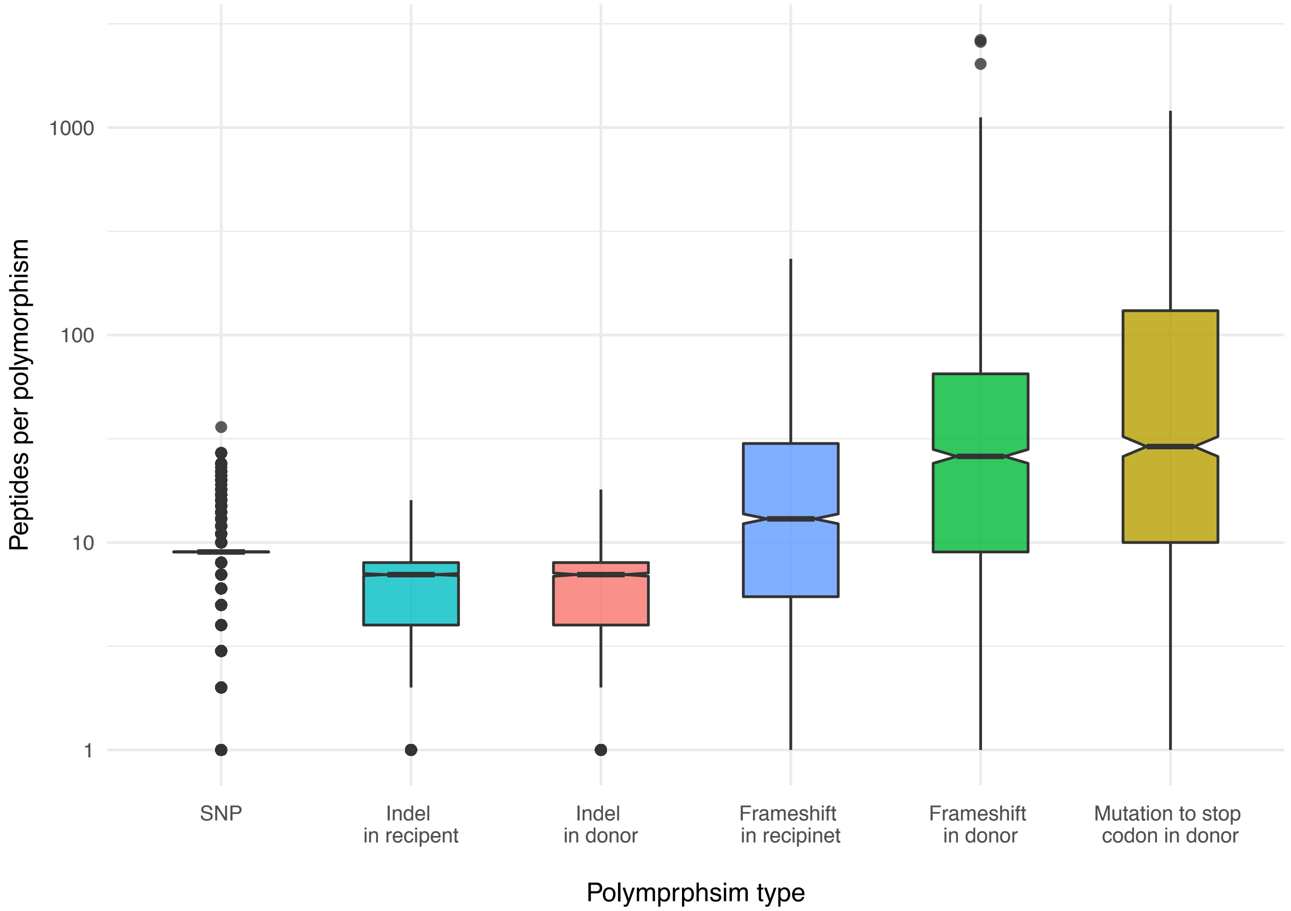

Supplementary Figure 2. The number of URPs per polymorphism. The distribution of the number of URPs caused by a particular polymorphism in either donor or the recipient is shown in the form of boxplot.

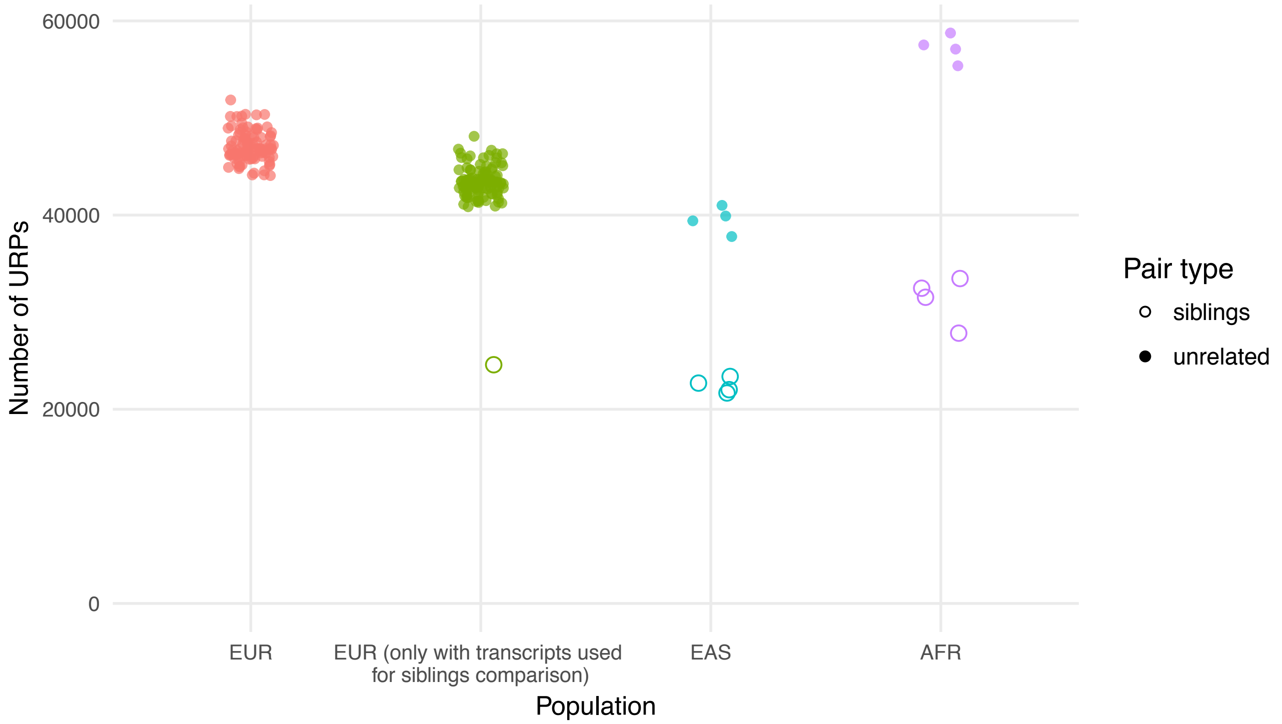

Supplementary Figure 3. The number of URPs in sibling vs. unrelated pairs. The pairs are divided by population group. Sibling pairs are shown by empty circles, unrelated pairs are shown by solid circles. For the European population all 100 unrelated pairs used in the paper are shown, while only one sibling pair was available. For EAS and AFR population 4 sibling pairs were available. Unrelated pair for one of the siblings was selected randomly. For the comparison of sibling and unrelated pairs a restricted set of transcripts was used due to the different release versions of the data (see Materials and Methods), all data except red circles.

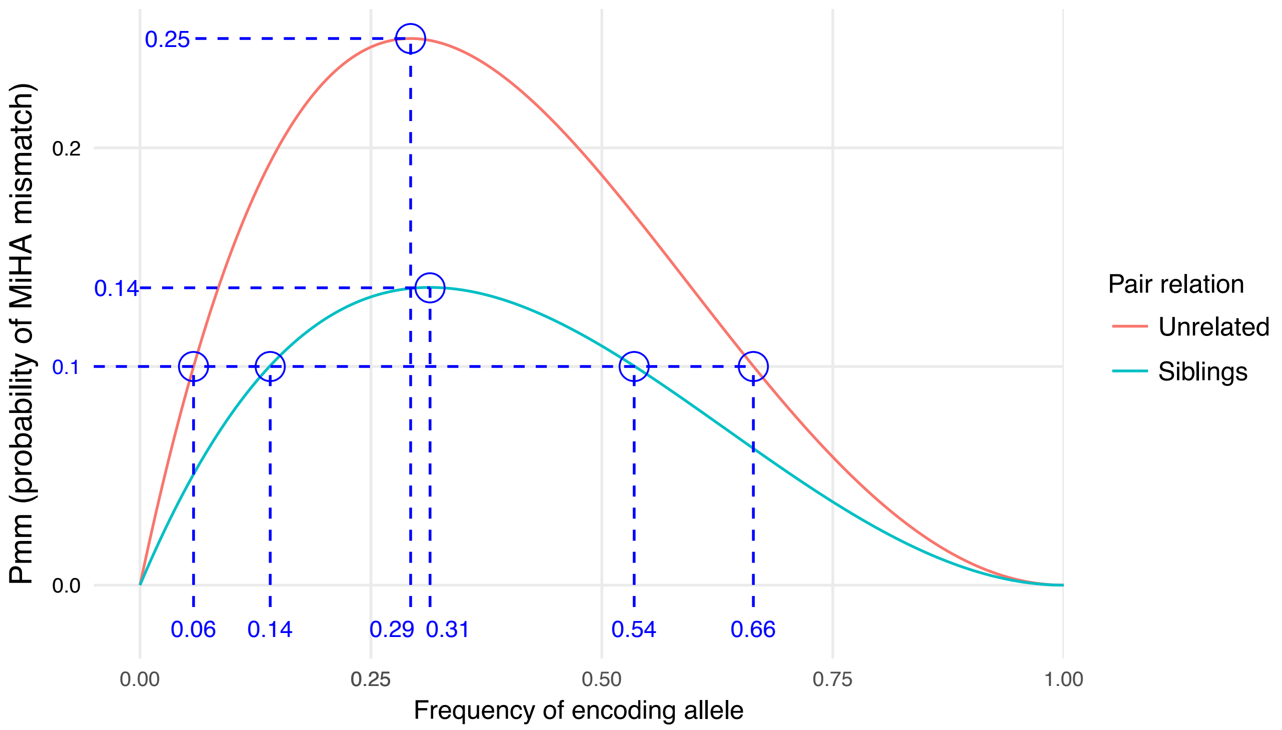

Supplementary Figure 4. The theoretical curve for the probability of URP occurrence for unrelated and sibling pairs. Dashed lines intersections show maximal values of Pmm (probability of MiHA mismatch), Pmm equal to 10%, and the allelic frequencies at which this Pmm values are achieved.

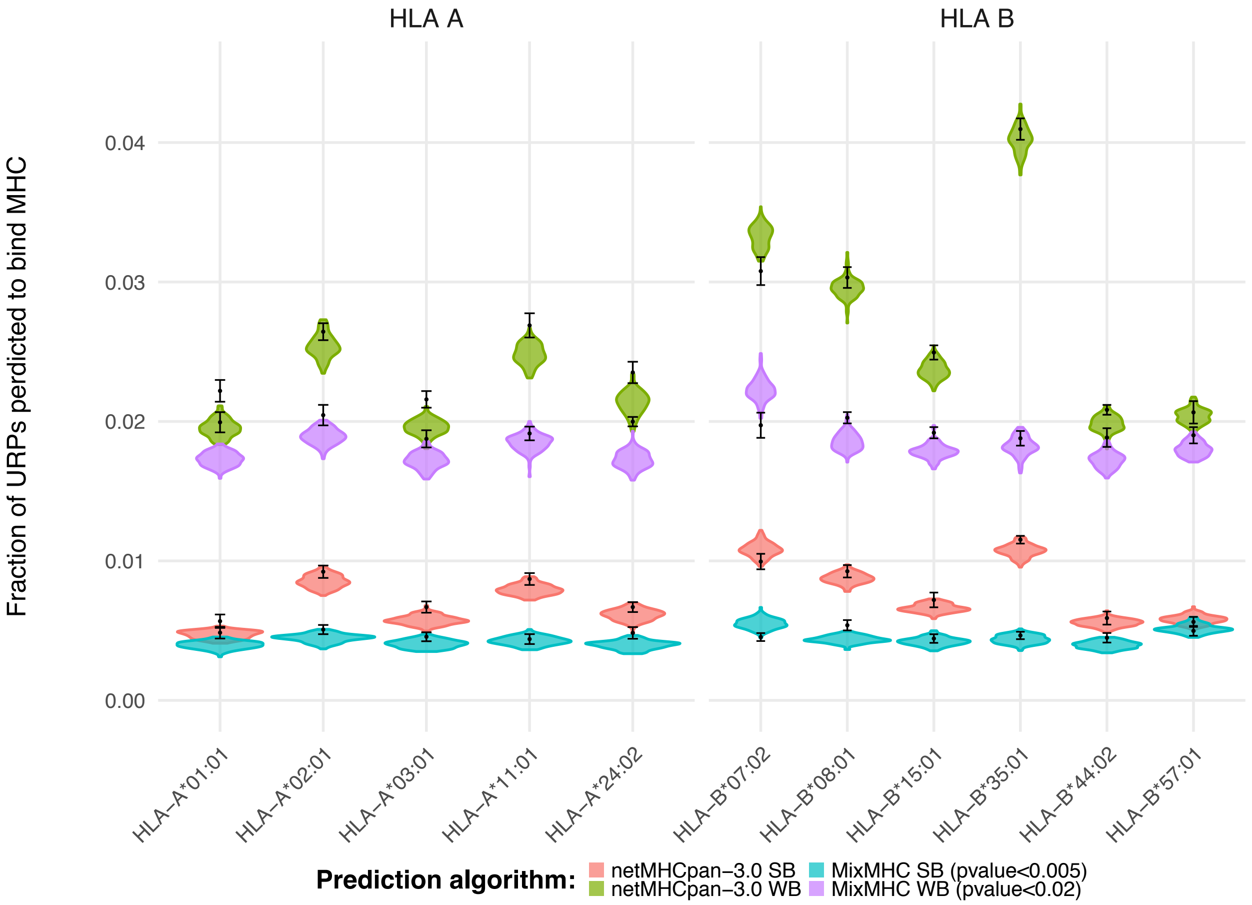

Supplementary Figure 5. The fraction of URPs predicted to bind MHC. The proportion of URPs predicted to bind MHC is shown for most frequent HLA alleles and variance among pairs is depicted with violin density plots. The results of different prediction programs and different prediction thresholds are shown with color, SB - strong binders, WB - weak binders. The black dots with error bars depicts the same value calculated for the random samples of peptides selected from the reference peptidome (the size of the random sample is equal to the average size of URPs in a pair).

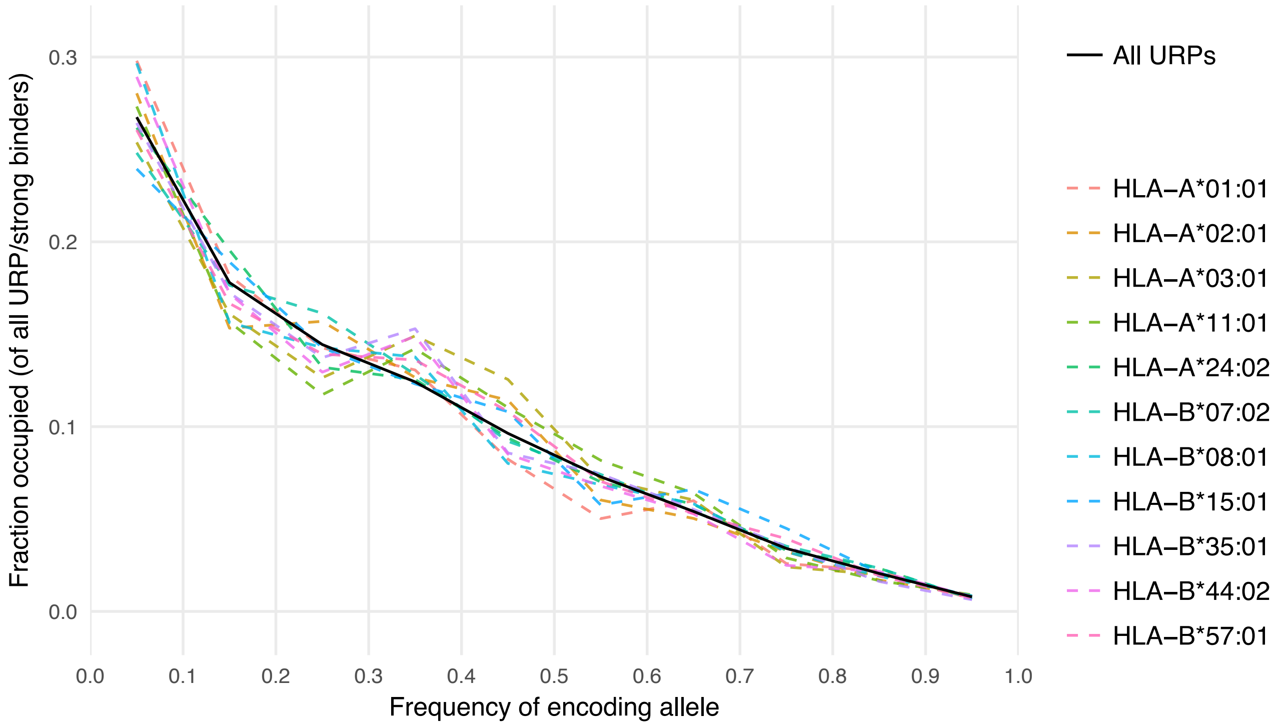

Supplementary Figure 6. The distribution of URPs and URiPs by the frequency of encoding allele. The distribution for URPs is shown in black solid line, dashed lines represent distributions of strong binder URiPs predicted for various HLA alleles. The data is averaged among all considered pairs.
