# Supplementary material for: In Silico Analysis of the Minor Histocompatibility Antigen Landscape Based on the 1000 Genomes Project
Source: Front Immunol. 2018 Aug 16;9:1819. doi: 10.3389/fimmu.2018.01819 (PMC6105694; doi:10.3389/fimmu.2018.01819)
Supplement: Supplementary file 7 [file data_sheet_2.PDF]

frontiers

# Supplementary Material

# *In Silico* Analysis of the Minor Histocompatibility Antigen Landscape Based on 1000 Genomes Project

**Nadia A. Bykova [1], Dmitry B. Malko [1], Grigory A. Efimov[1*]**

[1]Laboratory of Transplantation Immunology, National Research Center for Hematology, Moscow, Russia

*** Correspondence:**

Nadia Bykova 4noodle@gmail.com
Grigory Efimov , PhD, MD efimov.g@blood.ru

## 1 Supplementary Text 1. Derivation of the formula for the probability of MiHA mismatch.

### 1.1 For unrelated pairs

We denote the frequency of MiHA-encoding allele as *f*. Then, in the case of bi-allelic locus and Hardy-Weinberg assumptions, the frequency of the alternative allele is *(1-f)*, the probability of an individual to be homozygous by MiHA-encoding allele $P_{+/+} = f^2$, the probability to be homozygous by the alternative allele is $P_{-/-} = (1-f)^2$, and the probability to be heterozygous at this locus $P_{+/-} = 2\times f\times(1-f)$. The MiHA mismatch is defined as a situation, when the donor does not bear MiHA-encoding allele (i.e. homozygous by alternative allele), and the recipient bears at least one MiHA-encoding allele (i.e. homozygous by MiHA-encoding allele or heterozygous). Accordingly, the probability to observe a given MiHA mismatch in a pair is defined by the formula:

$$P_{mm} = P_{-/-}\times(P_{+/+} + P_{+/-}) = f\times(1-f)^2\times(2-f),$$

where *f* is the frequency of MiHA-encoding allele.

### 1.2 For sibling pairs

Let us consider two unrelated parents and estimate the probability of their sibling offspring to be mismatched at specific MiHA. The frequency of the three individual MiHA genotypes are described in the upper section. Accordingly, for a pair of individuals there exist 6 combinations of pairing, i.e. 6 variants of genotype pairs. The frequency of each pair genotype in the population is as follows:

1) Parent genotype 1: $P_{pair_1} = P_{(+/+,+/+)} = f^4$
2) Parent genotype 2: $P_{pair_2} = P_{(+/+,-/-)} = 2\times f^2\times(1-f)^2$
3) Parent genotype 3: $P_{pair_3} = P_{(+/+,+/-)} = 2\times f^2\times 2\times f\times(1-f)$
4) Parent genotype 4: $P_{pair_4} = P_{(+/-,+/-)} = 2\times f\times(1-f)\times 2\times f\times(1-f)$
5) Parent genotype 5: $P_{pair_5} = P_{(+/-,-/-)} = 2\times 2\times f\times(1-f)\times(1-f)^2$
6) Parent genotype 6: $P_{pair_6} = P_{(-/-,-/-)} = (1-f)^4$

The sum of all the 6 terms equals 1. Let us now consider the probability of MiHA mismatch between offspring for each of the cases:

1) All the offspring bear MiHA-encoding allele. $P_{mm1} = 0$ due to the absence of the donor homozygous by alternative allele.
2) All the offspring bear MiHA-encoding allele, $P_{mm2} = 0$.
3) All the offspring bear MiHA-encoding allele (from the +/+ parent), $P_{mm3} = 0$.
4) The probabilities of offspring genotypes: $P^{O4}_{(+/+)} = 1/4$, $P^{O4}_{(+/-)} = 1/2$, $P^{O4}_{(-/-)} = 1/4$. $P_{mm4} = P^{O4}_{(-/-)} \times (P^{O4}_{(+/+)} + P^{O4}_{(+/-)}) = 1/4 \times 3/4 = 3/16$.
5) The probabilities of offspring genotypes: $P^{O5}_{(+/-)} = 1/2$, $P^{O5}_{(-/-)} = 1/2$. $P_{mm5} = P^{O5}_{(-/-)} \times P^{O5}_{(+/-)} = 1/2 \times 1/2 = 1/4$.
6) All the offspring is equal and homozygous by alternative allele. $P_{mm6} = 0$ due to the absence of recipient with MiHA-encoding allele.

The total probability of MiHA-mismatch between siblings is given as a sum of such probabilities for each case given the frequency of the case in the population:

$P_{mm} = \sum_i \left(P_{pair_i} \times P_{mm_i}\right) = 0 \times P_{(+/+,+/+)} + 0 \times P_{(+/+,-/-)} + 0 \times P_{(+/+,+/-)} + 3/16 \times P_{(+/-,+/-)} + 1/4 \times P_{(+/-,-/-)} + 0 \times P_{(-/-,-/-)} = f \times (1-f)^2 \times \left(3/4 f + (1-f)\right) = f \times (1-f)^2 (4-f)/4$.
